# Class IIa Histone Deacetylases Are Conserved Regulators of Circadian Function*

**DOI:** 10.1074/jbc.M114.606392

**Published:** 2014-09-30

**Authors:** Paul C. M. Fogg, John S. O'Neill, Tomasz Dobrzycki, Shaun Calvert, Emma C. Lord, Rebecca L. L. McIntosh, Christopher J. H. Elliott, Sean T. Sweeney, Michael H. Hastings, Sangeeta Chawla

**Affiliations:** From the ‡ Department of Biology, University of York, York YO10 5DD, United Kingdom and; the § Medical Research Council Laboratory of Molecular Biology, Francis Crick Avenue, Cambridge CB2 0QH, United Kingdom

**Keywords:** Circadian Rhythm, Clock Gene, Drosophila, Histone Deacetylase (HDAC), Nuclear Translocation

## Abstract

Class IIa histone deacetylases (HDACs) regulate the activity of many transcription factors to influence liver gluconeogenesis and the development of specialized cells, including muscle, neurons, and lymphocytes. Here, we describe a conserved role for class IIa HDACs in sustaining robust circadian behavioral rhythms in *Drosophila* and cellular rhythms in mammalian cells. In mouse fibroblasts, overexpression of HDAC5 severely disrupts transcriptional rhythms of core clock genes. HDAC5 overexpression decreases BMAL1 acetylation on Lys-537 and pharmacological inhibition of class IIa HDACs increases BMAL1 acetylation. Furthermore, we observe cyclical nucleocytoplasmic shuttling of HDAC5 in mouse fibroblasts that is characteristically circadian. Mutation of the *Drosophila* homolog *HDAC4* impairs locomotor activity rhythms of flies and decreases *period* mRNA levels. RNAi-mediated knockdown of *HDAC4* in *Drosophila* clock cells also dampens circadian function. Given that the localization of class IIa HDACs is signal-regulated and influenced by Ca^2+^ and cAMP signals, our findings offer a mechanism by which extracellular stimuli that generate these signals can feed into the molecular clock machinery.

## Teaser

**Background:** Class IIa HDACs are signal-dependent transcriptional corepressors that regulate cell differentiation programs and liver gluconeogenesis.

**Results:** HDAC5 influences BMAL1 acetylation and interfering with normal expression levels of class IIa HDACs disrupts circadian rhythms.

**Conclusion:** Class IIa HDACs regulate the robustness of cellular clocks and behavioral activity rhythms.

**Significance:** Class IIa HDACs provide a conserved link between circadian clocks and metabolic signaling pathways.

## Introduction

Circadian rhythms govern diurnal variations in physiological functions, synchronizing behavior to the 24-h cyclical changes in our environment. At the molecular level, circadian clocks involve periodic changes in gene expression achieved by transcription-translation feedback loops whereby the protein products of transcribed genes auto-regulate their own transcription. In mammals, the core transcriptional circuit is comprised of the transcription factors CLOCK and BMAL1 that heterodimerize and activate transcription of Period (*Per*) and Cryptochrome (*Cry*) genes. PER/CRY proteins then repress their own transcription by inhibiting the activity of CLOCK-BMAL1 until they are degraded to allow a new cycle of transcription to begin (1). In addition, an interlocking feedback loop regulates rhythmic expression of *Bmal1* through opposing actions of the ROR and REV-ERB families of orphan nuclear receptors that activate and repress *Bmal1* transcription, respectively, and whose expression is controlled by the core loop (1–3). This mechanism is conserved in the *Drosophila* core loop, where heterodimers of CLOCK and CYCLE induce transcription of *period* and *timeless* and the interlocking loop generates rhythmic changes in *clock* expression (4). These transcriptional oscillations are regulated by many post-translational events, including reversible protein acetylation that controls circadian gene expression by impinging on both transcription factor activity and chromatin structure via modification of histone proteins. Rhythmic histone acetylation has been observed at promoters of core clock genes (5) and at promoters of clock-controlled output genes (6). Additionally, many core components of the molecular clock, including BMAL1 and PER2, show daily oscillations in their acetylation status (7, 8). These rhythms in acetylation are generated by cellular histone acetyltransferases and histone deacetylases (HDACs).^3^ CLOCK-BMAL1 heterodimers recruit the transcriptional coactivators p300 and CREB-binding protein, which possess histone acetyltransferase activity (5, 9). Moreover, CLOCK itself has been reported to possess intrinsic histone acetyltransferase activity (10). In mammals, SIRT1 has been implicated in opposing the activity of histone acetyltransferases to regulate rhythmic acetylation of BMAL1 (7), PER2 (8), and histone H3 (8) in response to cellular energy levels. Class IIa histone deacetylases are related HDACs whose subcellular localization is regulated by extracellular stimuli via the second messengers Ca^2+^ and cAMP (11). In fact, many SIRT1 substrates also interact with class IIa HDACs. For example, in response to nutrients SIRT1 deacetylates FOXO (12) but in response to hormone signaling, FOXO deacetylation is mediated by interactions with class IIa enzymes (13, 14). Class IIa HDACs and SIRT1 both interact with MEF2 transcription factors (15) and HIC-1 (hypermethylated in cancer 1; 16) to coordinate their deacetylation and SUMOylation.

Mammalian class IIa HDACs lack intrinsic enzymatic activity and instead mediate deacetylation of proteins via recruitment of corepressor complexes containing HDAC3, a class I HDAC, and the nuclear receptor corepressors NCoR and SMRT (silencing mediator of retinoic and thryoid hormone receptors) (17). For example, HDAC4 recruits the nuclear corepressor NCoR and HDAC3 to deacetylate FOXO transcription factors (14). The recruitment of SMRT/NCoR-HDAC3 complexes by class IIa HDACs could also affect histones and influence chromatin (18). Given that class IIa HDACs have the potential to influence rhythms of gene expression through their effects on both histones and non-histone proteins, we investigated their role in circadian function.

## EXPERIMENTAL PROCEDURES

### 

#### 

##### Plasmids and Antibodies

Expression vectors for wild-type HDAC5-FLAG, wild-type HDAC5GFP (HDAC5^WT^), and GFP-fused HDAC5 mutant (HDAC5^MUT^) have been described previously (19). The luciferase reporter plasmids contain either the mouse *Bmal1* promoter (*Bmal1*::*luc*; 20) or the mouse *Per2* promoter (*Per2*::*luc*; 20) upstream of firefly luciferase gene. Plasmids encoding FLAG-tagged mouse CLOCK (FLAG-CLOCK) and Myc-tagged mouse BMAL1 (BMAL1-Myc) were kindly provided by Dr. Nicolas Cermakian (McGill University) and have been described (21). Primary antibodies against phospho-HDAC4/5 Ser-246/259, acetyl histone H3(K9), histone H3, and the FLAG epitope were obtained from Cell Signaling Technology. Anti-acetyl BMAL1(Lys-537) and anti-CLOCK were from Millipore, and anti-HDAC5 made in goat was purchased from Santa Cruz Biotechnology.

##### Cell Culture and Bioluminescence Measurements

NIH3T3 fibroblasts were maintained in DMEM supplemented with 10% fetal bovine serum (FBS), 1% Glutamax, and penicillin/streptomycin (all from Invitrogen). Cells were transfected 24 h after plating using Gene Juice (Novagen, San Diego, CA) according to the manufacturer's instructions. Plasmids were transfected with the indicated expression constructs in a ratio 7:2:1 expression vector:circadian (firefly) luciferase reporter:pRL-CMV (*Renilla* luciferase, Promega). *Renilla* luciferase activity was used as an internal control to correct for transfection efficiency. Cells were synchronized by replacing the medium with air medium and sealing the dishes prior to bioluminescence recordings, which were performed using custom-made photomultiplier assemblies housed in a 37 °C incubator as described previously (22).

##### Drosophila Stocks and Behavioral Assays

All fly stocks were maintained on standard yeast-sugar-agar food. The *HDAC4^KG09091^* hypomorph mutant (13) was obtained from the Bloomington Stock Center (Indiana University). *UAS-HDAC4^RNAi^* (VDRC 20522) strain was obtained from the Vienna *Drosophila* RNAi Center (Vienna, Austria). The *y w;tim-Gal4* driver line (23) was obtained from Professor Ralf Stanewsky (Queen Mary, University of London). A DAM2 *Drosophila* activity monitor system (Trikinetics, Inc., Waltham, MA) was used to record locomotor activity in 2-min bins. 1-to-4-day-old adult males were collected and loaded into activity tubes containing 5% sucrose in 1% agar food at one end. Flies were entrained to 12-h light/12-h dark cycles (LD) at 25^°^C for 3 days and then monitored in constant darkness for 7 to 10 days. Activity records were analyzed using ActogramJ (24), and circadian rhythmicity was assessed by Lomb-Scargle periodogram analysis of the constant darkness data.

##### Drosophila RNA Analysis

2-to-4-day-old males were entrained for 3 days in LD conditions and then frozen at the indicated zeitgeber times. Total RNA was extracted using TRIzol reagent (Invitrogen) and treated with DNase. 1 μg of RNA was reverse-transcribed with Superscript II reverse transcriptase (Invitrogen), and the resulting cDNA was amplified with gene-specific primers for semi-quantitative PCR analysis. Primer sequences for *Drosophila* genes were as follows: *period*, 5′-CGTGCTGTGTCTGGTCCTC-3′ (forward) and 5′-ACGGACAGCAATGGGAATAG-3′ (reverse); 18S rRNA, 5′-AACATGAACCTTATGGGACATGTG-3′ (forward) and 5′-TCGGTACAAGACCATACGATCTGC-3′ (reverse); and *HDAC4*, 5′-ACAACGCGTCCAGTAACTCC-3′ (forward) and 5′-CCAGTGTCGGGAATCTGACT-3′ (reverse).

##### Western Blotting

To assess the effect of HDAC inhibitors on BMAL1 acetylation, NIH3T3 cells transfected with BMAL1-Myc using FuGENE 6 (Roche Applied Science) were treated with indicated HDAC inhibitors 24 h after transfection. After 18 h, cells were lysed in hot SDS sample buffer. To assess the effect of HDAC5 expression on BMAL1 acetylation protein extracts were prepared from cells 40 h after transfection. Proteins were resolved by SDS-PAGE using pre-cast Bis-Tris gels (Invitrogen) and immunoblotted using standard protocols. Band intensities were quantified with ImageJ software. Statistical significance was determined with a two-tailed paired Student's *t* test using the SPSS Statistical suite (IBM).

##### HDAC5 Localization

HDAC5 subcellular localization was assessed by live cell imaging or immunofluorescence in near confluent cultures. For live cell imaging, HDAC5GFP-transfected NIH3T3 cells were imaged 24 h after transfection. Cells were imaged at 37 °C on a Zeiss laser scanning confocal system (Zeiss LSM 780 multiphoton) in medium (DMEM containing 10% FBS and penicillin/streptomycin) that was replaced immediately before the start of the experiment. Images of GFP transfected cells were captured every 2 h for 2 consecutive days. For immunofluorescence, NIH3T3 cells were fixed at indicated times after synchronization of cultures with 10 μm forskolin. Cells were fixed in 3% paraformaldehyde/4% sucrose in PBS for 20 min. Cells were subsequently washed in PBS followed by permeabilization in 0.5% Nonidet P-40 in PBS. Cells were incubated with phospho-HDAC4/5 antibody (diluted 1:100) overnight at 4^°^C. Alexa Fluor-conjugated secondary antibodies were used at 1:500 and nuclei were visualized with a Hoechst stain. Images were captured on a Zeiss laser scanning confocal system, and immunofluorescence intensities were assessed using ImageJ software.

## RESULTS

### 

#### 

##### Class IIa HDACs Regulate Normal Circadian Function

**FIGURE 1.**
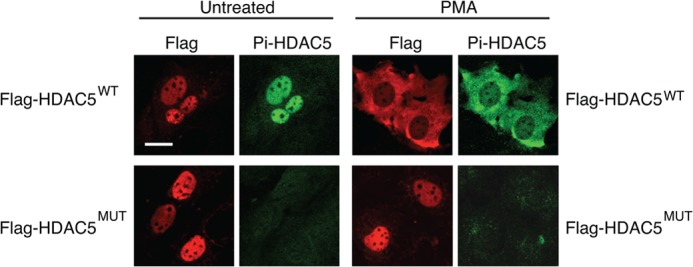
**Subcellular localization of wild-type HDAC5 and phosphorylation-defective HDAC5 mutant.** NIH3T3 cells were transfected with FLAG-tagged HDAC5 expression plasmids encoding either wild-type (FLAG-HDAC5^WT^; *top panel*) or mutant (S259A/S498A) HDAC5 (FLAG-HDAC5^MUT^; *bottom panel*). 24 h after transfection, cells were left untreated or stimulated with 200 nm phorbol 12-myristate 13-acetate (*PMA*) for 2 h and processed for immunofluorescence. HDAC5 expression was detected with a FLAG antibody, and HDAC5 Ser-259 phosphorylation was assessed using a phospho-HDAC4/5/7 antibody. Phospho-Ser-259 immunoreactivity co-localizes with HDAC5 and is detected in the nucleus of untreated FLAG-HDAC5^WT^ transfected cells. Phorbol 12-myristate 13-acetate stimulation induces HDAC5 nuclear export, and FLAG and phospho-HDAC5 immunoreactivity can be seen in the cytoplasm of FLAG-HDAC5^WT^ transfected cells. FLAG-HDAC5^MUT^ does not translocate to the cytoplasm in response to phorbol 12-myristate 13-acetate due to mutation of serines 259 and 498 and is not detected by the phospho-HDAC4/5/7 antibody. *Scale bar* is 10 μm.

**FIGURE 2.**
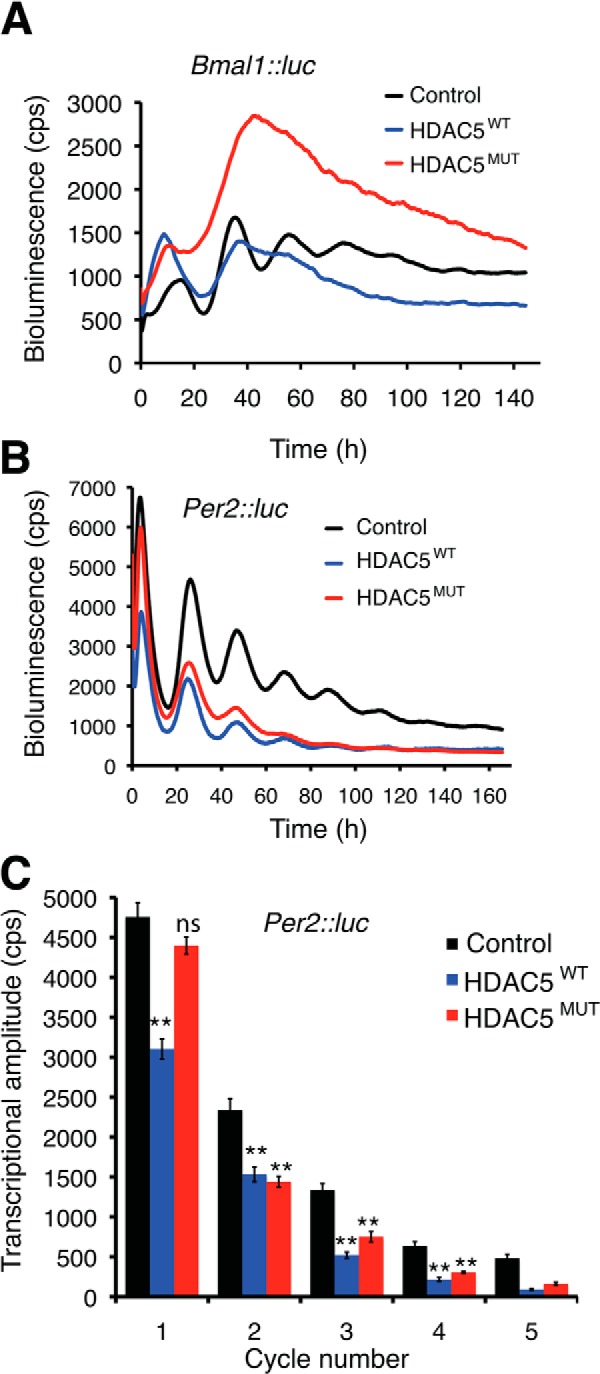
**HDAC5 overexpression compromises normal circadian function in NIH3T3 mouse fibroblasts.** *A*, representative traces of real-time bioluminescence from cells transfected with *Bmal1*::*luc* alone (control) or together with wild-type HDAC5GFP (HDAC5^WT^) or the constitutively nuclear mutant (HDAC5^MUT^). Representative results from six independent repetitions are shown. *B*, real-time bioluminescence oscillations from *Per2*::*luc* expressing fibroblasts in the absence (control) of HDAC5 or with co-expression of wild-type (HDAC5^WT^) or mutant (HDAC5^MUT^) HDAC5GFP. *C*, graph showing the effect of HDAC5^WT^ and HDAC5^MUT^ on the amplitude of *Per2*::*luc* oscillations over successive cycles. Data are from six biological replicates and shown as mean ± S.E. With the exception of the first cycle, there was no significant difference between HDAC5^WT^ and HDAC5^MUT^. *Double asterisks* indicate significant reduction compared with control; *p* < 0.01 (Student's *t* test). *ns* indicates no significant difference compared with the corresponding control.

Class IIa HDACs shuttle between the nucleus and the cytoplasm in response to extracellular signals. Their cytoplasmic localization is mediated by phosphorylation of two conserved serine residues in their N termini (serines 259 and 498 in HDAC5) that promotes their interaction with 14-3-3 proteins. To examine whether class IIa HDACs play a role in modulating circadian rhythms, we examined the effect of overexpressing both wild-type HDAC5 (HDAC5^WT^) and a phosphorylation-defective mutant (HDAC5^MUT^) (19) that is rendered constitutively nuclear due to mutation of the two conserved serines to alanines. Fig. 1 shows that the HDAC5^WT^ is nuclear in untreated NIH3T3 cells and translocates to the cytoplasm when cells are stimulated with phorbol 12-myristate 13-acetate, whereas HDAC5^MUT^ is constitutively nuclear. We examined the transcriptional rhythms of *Bmal1* and *Per2* in NIH3T3 mouse fibroblasts by co-expressing HDAC5 with luciferase reporter plasmids where the luciferase gene is driven either by the *Bmal1* promoter (*Bmal1*::*luc*) or the *Per2* promoter (*Per2*::*luc*). Real-time bioluminescence measurements show that HDAC5 overexpression severely compromised *Bmal1* transcriptional rhythms (Fig. 2*A*). Compared with control cells that displayed clear rhythmic *Bmal1* luciferase luminescence oscillations for four cycles, circadian rhythms were lost after the first cycle in cells expressing HDAC5^WT^ and completely abolished in cells expressing the constitutively nuclear HDAC5^MUT^ (Fig. 2*A*). Both HDAC5^WT^ and HDAC5^MUT^ dampened the amplitude of *Per2* transcriptional oscillations (Fig. 2, *B* and *C*) without affecting the period (unpaired *t* test compared with control, *p* > 0.5, *n* = 6). Expression of wild-type HDAC5 caused a significant reduction in the amplitude of *Per2* luciferase bioluminescence in the first cycle, whereas there was little effect of HDAC5^MUT^ expression. However, both the wild-type and mutant HDAC5 proteins caused a similar reduction in *Per2* luciferase amplitude and magnitude in successive cycles with an increased rate of dampening (Fig. 2*C*).

**FIGURE 3.**
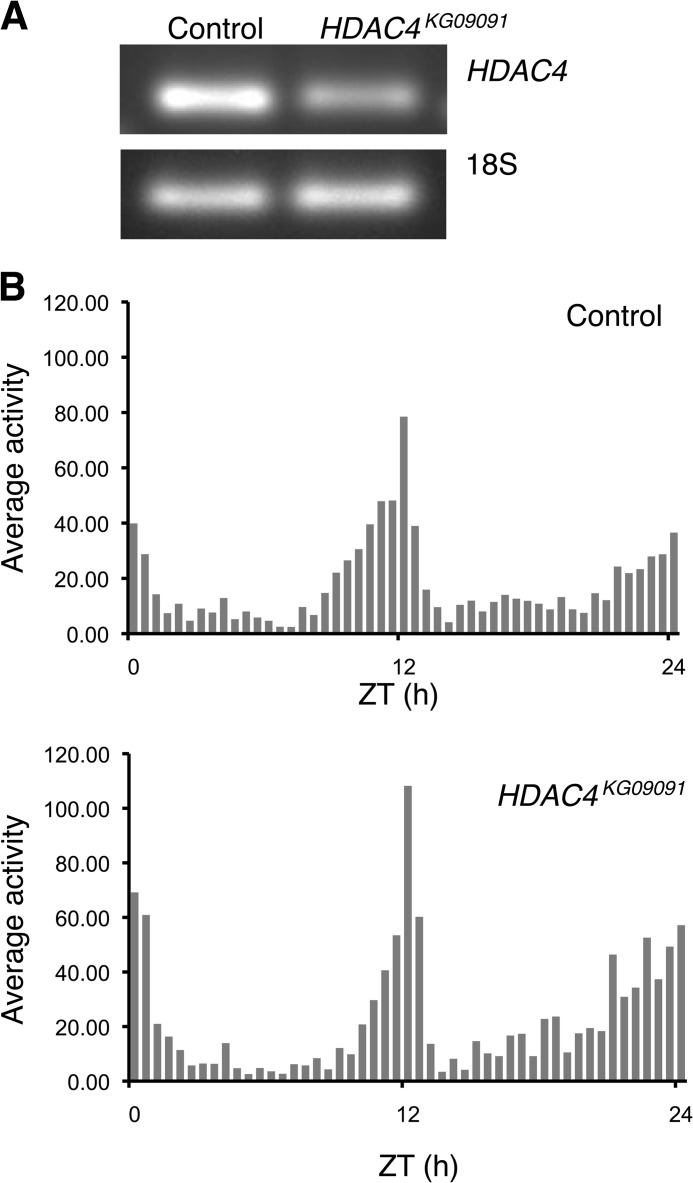
**HDAC4 mutant flies have reduced *HDAC4* mRNA levels and normal locomotor activity in light:dark cycles.** *A*, semi-quantitative RT-PCR of *HDAC4* mRNA levels and 18S rRNA levels in control (*ry506*/+) and *HDAC4^KG09091^* (*HDAC4^KG09091^/*+) mutant flies. *B*, average daily activity profiles of control and *HDAC4^KG09091^* mutant flies at indicated zeitgeber times (*ZT*) calculated from the final 3 days of LD cycles in 30-min bins (*n* = 15 flies). RNA analysis and activity profiles are of male flies produced by crossing *ry506* or *HDAC4^KG09091^* virgins with *Canton-S* male flies.

**FIGURE 4.**
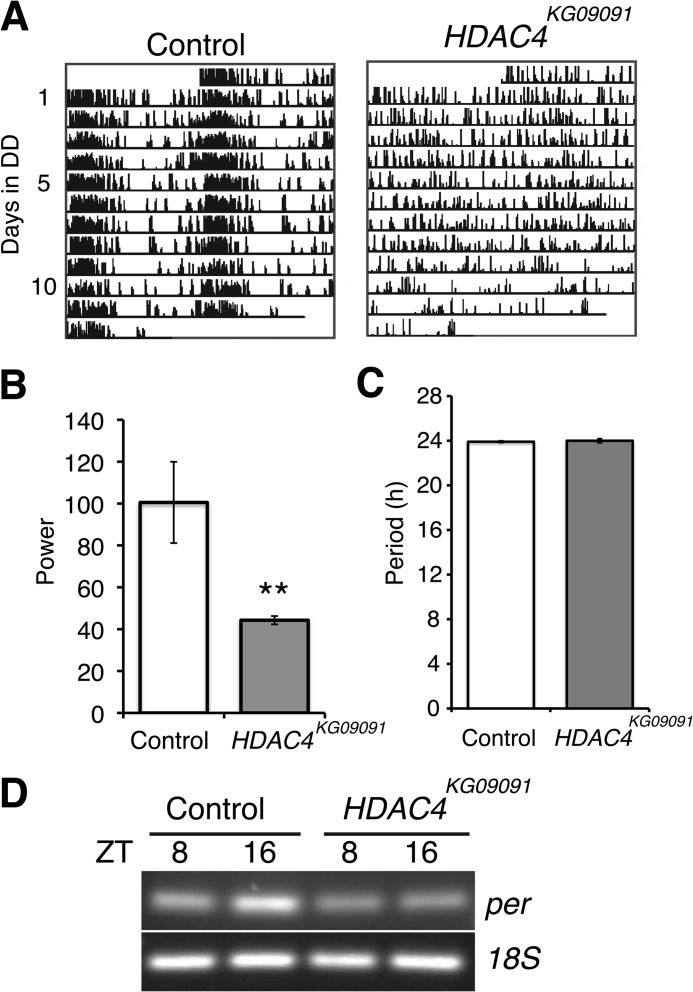
**Impaired circadian function in *Drosophila* lines with reduced levels of HDAC4 expression.** *A*, representative double-plotted actograms of control and *HDAC4^KG09091^* mutant male flies in constant darkness (*DD*). Control flies were generated by crossing *ry506* virgins with *Canton-S* males, and test flies were produced by crossing *HDAC4^KG09091^* virgins with *Canton-S* males. *B*, the strength of circadian activity rhythms is significantly reduced in *HDAC4^KG09091^* mutant flies. The power was calculated from a Lomb-Scargle periodogram analysis of 46 flies for each genotype. 41.3% of *HDAC4^KG09091^* flies were arrhythmic so the power was calculated from analyses of flies with rhythmic locomotor activity profiles. Data are shown here as the mean ± S.E. (**, *p* < 0.01 two-tailed Student's *t* test). *C*, graph showing the average period (± S.E.) of control and *HDAC4^KG09091^* mutant flies with rhythmic locomotor activity profiles. *D*, semi-quantitative PCR analysis of *period* (*per*) mRNA and 18S ribosomal RNA levels in control and *HDAC4^KG09091^* mutant flies. Flies were collected at the indicated *zeitgeber* times (*ZT*) after 3 days in LD.

We next investigated whether class IIa HDACs play a conserved role in sustaining normal circadian rhythms. Because many components of the core transcriptional-translational feedback loop are conserved in *Drosophila*, we examined locomotor activity rhythms in *Drosophila* lines with reduced expression of the fly homolog *HDAC4*. We used a HDAC4 hypomorph mutant line (*HDAC4^KG09091^*) containing a P-element insertion in the 5′-UTR, which leads to reduced *HDAC4* mRNA levels (Fig. 3*A*). We assessed locomotor activity in *HDAC4^KG09091^* flies that were out-crossed with the wild-type strain, *Canton-S* and compared these with control flies generated by crossing the *ry506* strain with *Canton-S*. The mutant flies showed normal locomotor activity profiles and circadian rhythms in 12-h LD cycles (Fig. 3*B*). However, in free-running conditions of constant darkness, 41% of HDAC4 mutant flies lacked any rhythms. Fig. 4*A* shows representative actograms of arrhythmic HDAC4 mutant flies and control flies. Lomb-Scargle periodogram analysis revealed that the remaining HDAC4 mutant flies that showed rhythmic behavior exhibited weak rhythms with a 60% decrease (Fig. 4*B*) in the power (a measure of the strength) of the rhythm. Although HDAC4 mutants displayed less robust rhythms, there was no significant difference in the period (Fig. 4*C*). To investigate whether *HDAC4* knockdown in *Drosophila* affects the molecular clock that underlies behavioral rhythms, we tested the effects of *HDAC4* gene disruption on *period* mRNA levels. HDAC4 mutant flies had reduced *period* mRNA expression in LD cycles at ZT16 but had comparable *period* expression at ZT8 (Fig. 4*D*).

Because HDAC4 is involved in many functions, including differentiation of *Drosophila* muscle, we used the Gal4/UAS system to direct RNAi-mediated knockdown of *HDAC4* expression. We crossed transgenic flies that express RNAi directed against *HDAC4* driven by a UAS element (*UAS-HDAC4^RNAi^*) with those that express Gal4 driven by the *timeless* gene promoter (*tim-Gal4*) to specifically knockdown *HDAC4* RNA levels in all clock cells. Table 1 shows that *Drosophila* with reduced expression of *HDAC4* in clock cells have either disrupted rhythms (31% arrhythmic flies) or weaker circadian activity rhythms, but the period remains unaffected. This is consistent with the behavioral phenotype of *HDAC4^KG09091^* mutant flies. Taken together, our experiments in mammalian cells and *Drosophila* lines indicate that interfering with normal levels of class IIa HDAC expression disrupts circadian function at both the cellular and behavioral level.

**TABLE 1:** **HDAC4 knockdown reduces stringency of *Drosophila* locomotor activity rhythms** Average period and the power of rhythms was deduced from Lomb-Scargle periodogram analysis of rhythmic flies. At least 16 flies of each genotype were analyzed, and values are shown as mean ± S.E.

Genotype	% Arrhythmic	Period	Power	*n*
		*h*		
*ry506*/+	0.0	24.3 ± 0.06	102 ± 19.4	46
*HDAC4*^KG09091^/+	41.3	24.2 ± 0.23	44.2 ± 2.0	46
*UAS-HDAC4^RNAi^*/+	0.0	24.4 ± 0.11	170.6 ± 21.4	16
*tim-Gal4*/+	0.0	23.8 ± 0.06	106.6 ± 16.8	16
*UAS-HDAC4^RNAi^*/*tim-Gal4*	31.2	24.2 ± 0.17	69.7 ± 7.9	16
*Canton-S*	4.0	24.5 ± 0.09	96.5 ± 10.2	25
*w^1118^*	6.2	24.6 ± 0.30	115.8 ± 28.9	16

##### HDAC5 Influences BMAL1 Acetylation

**FIGURE 5.**
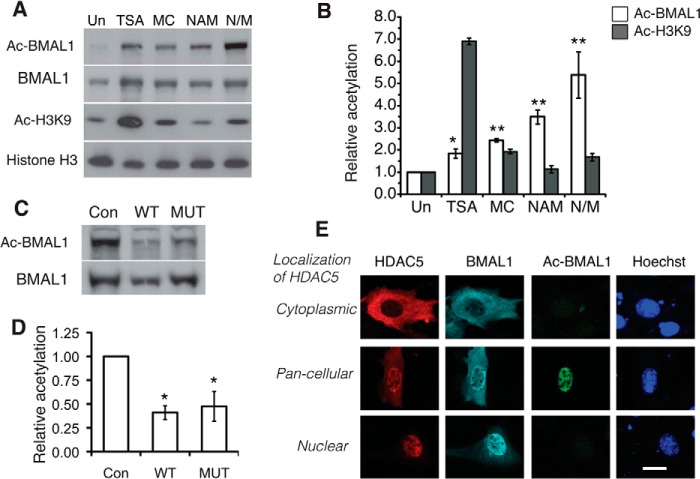
**HDAC5 influences BMAL1 acetylation and interacts with CLOCK-BMAL1.** *A*, representative Western blot showing the effect of HDAC inhibitors on BMAL1 acetylation at Lys-537 and H3 acetylation at Lys-9. 24 h after transfection, cells were left untreated (*Un*) or incubated with indicated HDAC inhibitors: 2 μm trichostatin A (*TSA*) or 10 μm MC1568 (*MC*), or 10 mm NAM, or 10 μm MC1568 and 10 mm NAM together (*N/M*). After 18 h, cells were processed for immunoblotting using anti-acetyl Lys-537 BMAL1 antibody. Immunoblots were stripped and sequentially probed with an antibody to Myc, followed by an anti-acetyl H3 and finally an anti-histone H3 antibody. *B*, quantitative analysis of BMAL1 acetylation and H3K9 acetylation from immunoblots in *A* is shown in the graph. The immunoreactivity was measured using ImageJ software, and acetyl-BMAL1 immunoreactivity was normalized to the corresponding BMAL1-Myc level, and H3K9 immunoreactivity was normalized to that of H3. Values are shown as mean ± S.E., and data are from three independent experiments. *Double asterisks* indicate significant increase in BMAL1 acetylation when compared with the control (*Un*); *p* < 0.01 (Student's *t* test). A *single asterisk* indicates significant increase in BMAL1 acetylation when compared with the control (*Con*), *p* < 0.05 (Student's *t* test). *C*, representative Western blot showing the effect of HDAC5^WT^ and HDAC5^MUT^ expression on BMAL1 Lys-537 acetylation. *D*, graph showing quantitative analysis of immunoblots in *C*. Data are from four independent transfection experiments and is shown as mean ± S.E. A *single asterisk* indicates significant reduction in BMAL1 acetylation when compared with the control *p* < 0.05 (Student's *t* test) *E*, representative examples of cells showing localization of HDAC5-FLAG and BMAL1-Myc when co-expressed. BMAL1 acetylation was assessed in cotransfected cells, and the nucleus is identified by Hoechst staining. *Scale bar* is 10 μm.

We next investigated whether class IIa HDACs can, similar to SIRT1, influence acetylation of BMAL1. We treated BMAL1-Myc transfected NIH3T3 cells with various HDAC inhibitors and assessed BMAL1 acetylation using an antibody specific for lysine 537-acetylated BMAL1. Trichostatin A, a class I and class II HDAC inhibitor increased BMAL1 acetylation levels 1.85-fold (Fig. 5, *A* and *B*). Trichostatin A had a dramatic effect on histone H3 Lys-9 acetylation, increasing it by 6.9-fold, a consequence of its inhibitory action on class I HDACs. The class II-specific HDAC inhibitor MC1568 (25) also increased BMAL1 acetylation, and the effect was similar to the class III HDAC inhibitor nicotinamide (NAM). MC1568 and NAM had an additive effect when applied together. MC1568 increased H3 Lys-9 acetylation to a lesser degree than trichostatin A, whereas NAM did not influence histone H3 acetylation. Although pharmacological inhibition of class II HDACs increased BMAL1 acetylation, we found that co-expression of either wild-type HDAC5 or the constitutively nuclear HDAC5 mutant with BMAL1-Myc decreased BMAL1 acetylation (Fig. 5, *C* and *D*). The wild-type and mutant HDAC5 were equally effective at deacetylating BMAL1.

The subcellular localization of BMAL1 changes during circadian cycles, and the nucleocytoplasmic shuttling of BMAL1 is abolished in fibroblasts with disrupted circadian clocks (26). We therefore examined the effect of HDAC5 co-expression on the subcellular localization of ectopically expressed BMAL1. In agreement with previous work (27), we found that BMAL1 was nuclear when expressed alone (data not shown). In contrast, the cellular localization of BMAL1 in HDAC5-expressing cells was heterogeneous, and BMAL1-Myc and HDAC5 could be found co-localized either in the cytoplasm (Fig. 5*E*, *top panel*), or in the nucleus (Fig. 5*E*, *bottom panel*), or in both the nucleus and cytoplasm (Fig. 5*E*, *middle panel*). Interestingly, when BMAL1 and HDAC5 were exclusively nuclear or exclusively cytoplasmic BMAL1 acetylation was abolished (Fig. 5 E, *top* and *bottom panel*). This suggests that cytoplasmically located wild-type HDAC5 prevents BMAL1 acetylation by sequestering it in the cytoplasm and nuclear HDAC5 (wild-type or mutant) likely recruits deacetylating enzymes such as HDAC3 to BMAL1. This would explain why wild-type and mutant HDAC5 influence BMAL1 acetylation to a similar extent. These data suggest that class IIa HDACs have a functional role in regulating BMAL1 acetylation.

##### HDAC5 Shows Circadian Changes in Subcellular Localization

**FIGURE 6.**
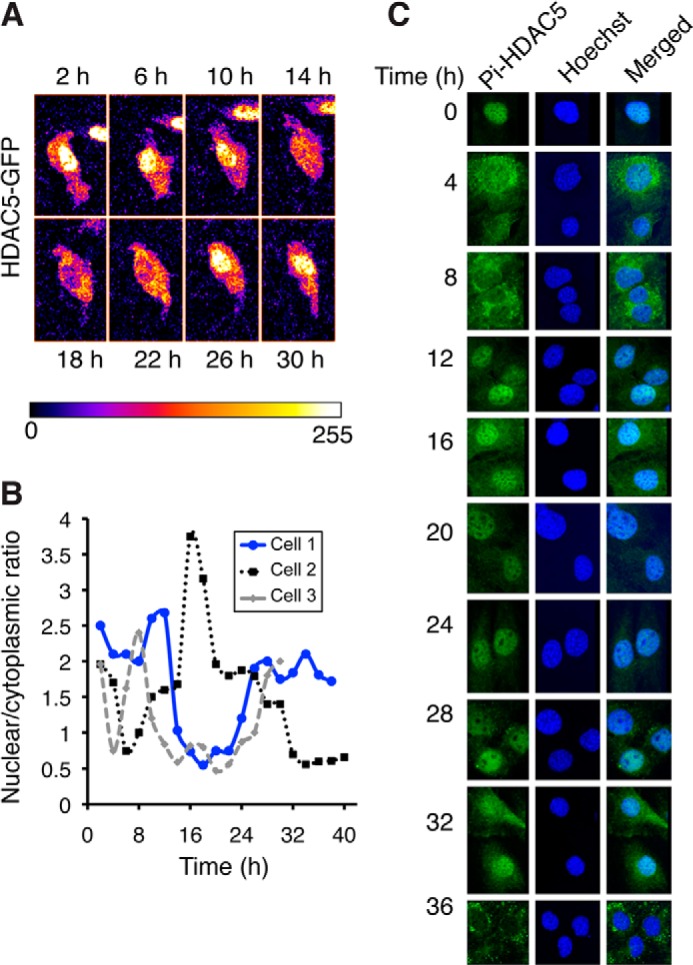
**HDAC5 subcellular localization displays circadian oscillations.** *A*, HDAC5GFP nucleocytoplasmic shuttling visualized in live cells over time. Confocal images of near confluent NIH3T3 cells transfected with HDAC5GFP were captured every 2 h and are shown in a false color scale from 0 (minimum) to 255 (maximum). *B*, the ratio of HDAC5GFP nuclear and cytoplasmic fluorescence intensity in three individual cells was quantified and is shown in the graph. Images of cell 1 are shown in *A* above. *C*, representative examples of NIH3T3 cells showing phospho-HDAC5 Ser-259 immunofluoresence and Hoechst staining at indicated times after synchronization of cells with 10 μm forskolin.

BMAL1 acetylation has been shown to display a circadian profile (7). Our experiments above show that class IIa HDACs can influence BMAL1 acetylation but do not explain how HDAC5 might confer circadian periodicity to BMAL1 acetylation. Because class IIa HDACs shuttle in and out of the nucleus in a signal-dependent manner, we investigated whether HDAC5 shows oscillatory changes in its nuclear and cytoplasmic localization. To do this, we examined the localization of an HDAC5GFP fusion protein in live cells by time-lapse fluorescence microscopy. Images of transfected NIH3T3 cells were captured every 2 h, and the ratio of GFP fluorescence in the nucleus and cytoplasm was assessed. Fig. 6*A* shows an example of a typical cell in which HDAC5 shuttles in and out of the nucleus with a circadian profile. The nuclear/cytoplasmic ratio in this cell (cell 1, Fig. 6*B*) shows ∼24 h of cyclical changes. The nuclear/cytoplasmic ratio for two other cells is shown in the graph (Fig. 6*B*), with only partial synchronization being observed following media change, as was reported previously for uncoupled fibroblast bioluminescence rhythms (28). Because the nuclear export of HDAC5 has been reported to correlate with an increase in phosphorylation at serines 259 and 498, we used a phospho-Ser-259 specific antibody to examine whether the phosphorylation status of HDAC5 shows rhythmic changes and correlates with its cytoplasmic localization. Fig. 6*C* shows phospho-HDAC5 Ser-259 immunoreactivity in forskolin-synchronized NIH3T3 cells fixed every 4 h after synchronization. Surprisingly, phospho-Ser-259 immunoreactivity is detectable in the nucleus during this time course (Fig. 6*C*). Strikingly, phosphorylated HDAC5 shuttles in and out of the nucleus with a 24-h period: it is nuclear at time 0, but exclusively cytoplasmic at 8 h and again at 36 h after synchronization (Fig. 6*C*). To verify that the observed nuclear immunoreactivity detected with the phospho-Ser-259 is due to HDAC5, we validated that the antibody only recognizes expressed HDAC5^WT^, and no signal is detectable in cells transfected with the non-phosphorylatable mutant HDAC5^MUT^ (Fig. 1). The phospho-Ser-259 antibody detected both nuclear and cytoplasmic HDAC5^WT^ in NIH3T3 cells (Fig. 1). The changes in localization of HDAC5GFP in live cells and the temporal profile of phospho-Ser-259 HDAC5 immunoreactivity together indicate a cyclical variation in HDAC5 subcellular localization that does not depend on changes in Ser-259 phosphorylation. The time of day-dependent presence of HDAC5 in the nucleus could account for circadian changes in the acetylation status of BMAL1.

## DISCUSSION

Rhythmic changes in the acetylation status of core clock transcription factors and of histones at gene promoters play an important role in imparting circadian periodicity to physiological processes such as metabolism. In this context, the contribution of several histone acetyltransferases and HDACs has been studied. Among the HDAC family, the class I enzyme HDAC3 has been shown to control circadian expression of liver genes involved in lipid biosynthesis (6), and the class III NAD^+^-dependent enzyme SIRT1 modulates the core transcriptional oscillator in response to cellular energy levels (7, 8). In contrast to the reported effect of SIRT1 expression, which increased the amplitude of bioluminescence oscillations from *Bmal1*-luciferase and *Per2*-luciferase reporter genes in NIH3T3 cells (8); here, HDAC5 expression led to a loss of rhythmicity in *Bmal1*-luciferase oscillations and decreased the magnitude of *Per2*-luciferase bioluminescence oscillations. Thus, in mouse fibroblasts, HDAC5 expression appears to have a more severe circadian phenotype than SIRT1 expression. This is backed by differences in behavioral locomotor activity rhythms of HDAC4 and Sir2 mutant flies. We find impaired locomotor activity rhythms and lowered *period* expression in *Drosophila* HDAC4 mutants. Notably, we find that *Drosophila* lines with reduced expression of the SIRT1 homolog *Sir2* show no circadian defects in behavioral rhythms (data not shown). The dampened *Per2* transcriptional rhythms that persist with unaltered periodicity in the face of severely disrupted *Bmal1* transcriptional rhythms in HDAC5-transfected fibroblasts suggest that the ROR/REV-ERB-dependent interlocking feedback loop is not required for determining the period of circadian oscillations and is in agreement with previous work (3). Importantly, this accessory loop modulates the amplitude and magnitude of transcriptional rhythms in the core circuit, and this likely accounts for the less robust behavioral rhythms seen in *Drosophila* HDAC4 mutants.

At the molecular level, we find that HDAC4/5 inhibition led to increased acetylation of BMAL1 on Lys-537, and HDAC5 overexpression reduced BMAL1 acetylation. This effect of HDAC5 overexpression on BMAL1 acetylation would be expected to result in a loss of rhythmic expression of CLOCK/BMAL1-controlled target genes, including members of the REV-ERB and ROR family of proteins and could account for the deregulated *Bmal1* transcriptional rhythms seen in HDAC5-transfected cells. The acetylated residue in mouse BMAL1 (Lys-537) is highly conserved in vertebrates but is absent in the *Drosophila* homolog CYCLE, raising questions about how class IIa HDACs affect the molecular oscillator in *Drosophila*. It is possible that the observed acetylation/deacetylation of this residue in our HDAC5 inhibition/overexpression experiments reflects a loss of cellular rhythms mediated by molecular events unrelated to BMAL1 acetylation. Class IIa HDACs can act as E3 SUMO ligases and promote SUMOylation of several proteins. In this regard, it is noteworthy that mouse BMAL1 is SUMOylated on Lys-259, a residue that is conserved in *Drosophila* CYCLE (29). An interplay between HDAC4/5-mediated SUMOylation and SIRT1-mediated deacetylation has been described for MEF2 (15) and HIC1 (16). Interestingly, MEF2 proteins have been implicated in controlling circadian behavior in *Drosophila* (30). It is therefore likely that HDAC5 co-exists with SIRT1 in a regulatory complex with CLOCK-BMAL1 acting in concert to influence the core oscillator. Further studies will be required to illuminate the molecular basis for the conserved action of class IIa HDACs in mammalian and *Drosophila* clocks.

Signal-regulated nucleocytoplasmic shuttling is a distinct trait of class IIa HDACs that sets them apart from other HDAC family members. Here, we show that HDAC5 shows cyclical changes in its localization oscillating between the nucleus and cytoplasm with circadian timing. Of note, this nucleocytoplasmic shuttling appears to be independent of HDAC5 phosphorylation at the conserved serine 259. The antibody used here that detects phospho-HDAC5 Ser-259 would also recognize Ser-246 phosphorylated HDAC4, and it is possible that HDAC4 localization also changes with circadian timing. Depending on the cell type and the signal, phosphorylation of class IIa HDACs can be mediated by Ca^2+^/calmodulin-dependent kinases (CaMKII and CaMKIV), protein kinase D, salt-inducible kinases, and AMPK kinases and in most instances phosphorylation of HDAC5 on serines 259 and 498 leads to its nuclear exclusion (11). However, phospho-Ser-259-independent nuclear accumulation of HDAC5 has been reported in Cos7 cells where elevated levels of cellular cAMP inhibit phorbol 12-myristate 13-acetate-induced nuclear export of HDAC5 even when Ser-259 is phosphorylated (31). This raises the possibility that rhythmic changes in cellular cAMP levels, an integral feature of the core circadian oscillator (32), drive intrinsic oscillations in HDAC5 nucleocytoplasmic shuttling. In addition to regulating subcellular localization, phosphorylation of HDAC5 could also influence its interactions with other proteins such as class I HDACs. Sequential phosphorylations on distinct sites could regulate dissociation of HDAC5 from class I HDACs followed by 14-3-3 binding and subsequent nuclear export. This would explain why BMAL1 can be acetylated or deacetylated in the presence of wild-type nuclear HDAC5 (Fig. 5*E*, compare *middle* and *bottom rows*).

Nucleocytoplasmic shuttling of class IIa HDACs also occurs acutely in response to hormones and is involved in regulating gluconeogenesis in mammals (14) and in *Drosophila* (13). In the liver, the fasting hormone glucagon causes class IIa HDACs to accumulate in the nucleus where they deacetylate and activate FOXO transcription factors to induce expression of gluconeogenic genes. Insulin, on the other hand, promotes nuclear exclusion of HDAC4/5 by inducing their phosphorylation (13, 14). The self-sustained cell autonomous oscillations in HDAC5 localization that we have observed in NIH3T3 fibroblasts raise the possibility that the liver may be able to control glucose production with circadian periodicity in the absence of hormone signals or feeding-fasting cues. Our work thus adds class IIa HDACs to a growing list of proteins that are involved in mediating both evoked cellular changes in response to metabolic cues as well as sustaining cellular circadian cycles, allowing metabolic processes to feed back to the clock.

## Footnotes

3 The abbreviations used are:

HDAC: histone deacetylase
MEF2: myocyte enhancer factor-2
SUMO: small ubiquitin-like modifier
NAM: nicotinamide
LD: light/dark cycle.

## References

[1] Lowrey P. L. Takahashi J. S. (2011) Genetics of circadian rhythms in Mammalian model organisms. Adv. Genet. 74, 175–230 21924978 10.1016/B978-0-12-387690-4.00006-4 PMC3709251

[2] Preitner N. Damiola F. Lopez-Molina L. Zakany J. Duboule D. Albrecht U. Schibler U. (2002) The orphan nuclear receptor REV-ERBα controls circadian transcription within the positive limb of the mammalian circadian oscillator. Cell 110, 251–260 12150932 10.1016/s0092-8674(02)00825-5

[3] Liu A. C. Tran H. G. Zhang E. E. Priest A. A. Welsh D. K. Kay S. A. (2008) Redundant function of REV-ERBα and β and non-essential role for Bmal1 cycling in transcriptional regulation of intracellular circadian rhythms. PLoS Genet. 4, e1000023 18454201 10.1371/journal.pgen.1000023 PMC2265523

[4] Zheng X. Sehgal A. (2008) Probing the relative importance of molecular oscillations in the circadian clock. Genetics 178, 1147–1155 18385110 10.1534/genetics.107.088658 PMC2278066

[5] Etchegaray J. P. Lee C. Wade P. A. Reppert S. M. (2003) Rhythmic histone acetylation underlies transcription in the mammalian circadian clock. Nature 421, 177–182 12483227 10.1038/nature01314

[6] Feng D. Liu T. Sun Z. Bugge A. Mullican S. E. Alenghat T. Liu X. S. Lazar M. A. (2011) A circadian rhythm orchestrated by histone deacetylase 3 controls hepatic lipid metabolism. Science 331, 1315–1319 21393543 10.1126/science.1198125 PMC3389392

[7] Nakahata Y. Kaluzova M. Grimaldi B. Sahar S. Hirayama J. Chen D. Guarente L. P. Sassone-Corsi P. (2008) The NAD^+^-dependent deacetylase SIRT1 modulates CLOCK mediated chromatin remodeling and circadian control. Cell 134, 329–340 18662547 10.1016/j.cell.2008.07.002 PMC3526943

[8] Asher G. Gatfield D. Stratmann M. Reinke H. Dibner C. Kreppel F. Mostoslavsky R. Alt F. W. Schibler U. (2008) SIRT1 regulates circadian clock gene expression through PER2 deacetylation. Cell 134, 317–328 18662546 10.1016/j.cell.2008.06.050

[9] Takahata S. Ozaki T. Mimura J. Kikuchi Y. Sogawa K. Fujii-Kuriyama Y. (2000) Transactivation mechanisms of mouse clock transcription factors, mClock and mArnt3. Genes Cells 5, 739–747 10971655 10.1046/j.1365-2443.2000.00363.x

[10] Doi M. Hirayama J. Sassone-Corsi P. (2006) Circadian regulator CLOCK is a histone acetyltransferase. Cell 125, 497–508 16678094 10.1016/j.cell.2006.03.033

[11] Parra M. Verdin E. (2010) Regulatory signal transduction pathways for class IIa histone deacetylases. Curr. Opin. Pharmacol. 10, 454–460 20447866 10.1016/j.coph.2010.04.004

[12] Brunet A. Sweeney L. B. Sturgill J. F. Chua K. F. Greer P. L. Lin Y. Tran H. Ross S. E. Mostoslavsky R. Cohen H. Y. Hu L. S. Cheng H. L. Jedrychowski M. P. Gygi S. P. Sinclair D. A. Alt F. W. Greenberg M. E. (2004) Stress-dependent regulation of FOXO transcription factors by the SIRT1 deacetylase. Science 303, 2011–2015 14976264 10.1126/science.1094637

[13] Wang B. Moya N. Niessen S. Hoover H. Mihaylova M. M. Shaw R. J. Yates J. R. 3rd Fischer W. H. Thomas J. B. Montminy M. (2011) A hormone-dependent module regulating energy balance. Cell 145, 596–606 21565616 10.1016/j.cell.2011.04.013 PMC3129781

[14] Mihaylova M. M. Vasquez D. S. Ravnskjaer K. Denechaud P. D. Yu R. T. Alvarez J. G. Downes M. Evans R. M. Montminy M. Shaw R. J. (2011) Class IIa histone deacetylases are hormone-activated regulators of FOXO and mammalian glucose homeostasis. Cell 145, 607–621 21565617 10.1016/j.cell.2011.03.043 PMC3117637

[15] Zhao X. Sternsdorf T. Bolger T. A. Evans R. M. Yao T. P. (2005) Regulation of MEF2 by histone deacetylase 4- and SIRT1 deacetylase-mediated lysine modifications. Mol. Cell. Biol. 25, 8456–8464 16166628 10.1128/MCB.25.19.8456-8464.2005 PMC1265742

[16] Stankovic-Valentin N. Deltour S. Seeler J. Pinte S. Vergoten G. Guérardel C. Dejean A. Leprince D. (2007) An acetylation/deacetylation-SUMOylation switch through a phylogenetically conserved psiKXEP motif in the tumor suppressor HIC1 regulates transcriptional repression activity. Mol. Cell. Biol. 27, 2661–2675 17283066 10.1128/MCB.01098-06 PMC1899900

[17] Fischle W. Dequiedt F. Hendzel M. J. Guenther M. G. Lazar M. A. Voelter W. Verdin E. (2002) Enzymatic activity associated with class II HDACs is dependent on a multiprotein complex containing HDAC3 and SMRT/N-CoR. Mol. Cell 9, 45–57 11804585 10.1016/s1097-2765(01)00429-4

[18] Wen Y. D. Perissi V. Staszewski L. M. Yang W. M. Krones A. Glass C. K. Rosenfeld M. G. Seto E. (2000) The histone deacetylase-3 complex contains nuclear receptor corepressors. Proc. Natl. Acad. Sci. U.S.A. 97, 7202–7207 10860984 10.1073/pnas.97.13.7202 PMC16523

[19] Soriano F. X. Chawla S. Skehel P. Hardingham G. E. (2013) SMRT-mediated co shuttling enables export of class IIa HDACs independent of their CaM kinase phosphorylation sites. J. Neurochem. 124, 26–35 23083128 10.1111/jnc.12058 PMC3557716

[20] Sato T. K. Yamada R. G. Ukai H. Baggs J. E. Miraglia L. J. Kobayashi T. J. Welsh D. K. Kay S. A. Ueda H. R. Hogenesch J. B. (2006) Feedback repression is required for mammalian circadian clock function. Nat. Genet. 38, 312–319 16474406 10.1038/ng1745 PMC1994933

[21] Dardente H. Fortier E. E. Martineau V. Cermakian N. (2007) Cryptochromes impair phosphorylation of transcriptional activators in the clock: a general mechanism for circadian repression. Biochem. J. 402, 525–536 17115977 10.1042/BJ20060827 PMC1863574

[22] O'Neill J. S. Hastings M. H. (2008) Increased coherence of circadian rhythms in mature fibroblast cultures. J. Biol. Rhythms 23, 483–488 19060257 10.1177/0748730408326682 PMC2735814

[23] Kaneko M. Hall J. C. (2000) Neuroanatomy of cells expressing clock genes in *Drosophila*: transgenic manipulation of the period and timeless genes to mark the perikarya of circadian pacemaker neurons and their projections. J. Comp. Neurol. 422, 66–94 10842219 10.1002/(sici)1096-9861(20000619)422:1<66::aid-cne5>3.0.co;2-2

[24] Schmid B. Helfrich-Förster C. Yoshii T. (2011) A new ImageJ plug-in “ActogramJ” for chronobiological analyses. J. Biol. Rhythms 26, 464–467 21921300 10.1177/0748730411414264

[25] Nebbioso A. Manzo F. Miceli M. Conte M. Manente L. Baldi A. De Luca A. Rotili D. Valente S. Mai A. Usiello A. Gronemeyer H. Altucci L. (2009) Selective class II HDAC inhibitors impair myogenesis by modulating the stability and activity of HDAC MEF2 complexes. EMBO Rep. 10, 776–782 19498465 10.1038/embor.2009.88 PMC2693879

[26] Tamaru T. Isojima Y. van der Horst G. T. Takei K. Nagai K. Takamatsu K. (2003) Nucleocytoplasmic shuttling and phosphorylation of BMAL1 are regulated by circadian clock in cultured fibroblasts. Genes Cells 8, 973–983 14750952 10.1046/j.1365-2443.2003.00686.x

[27] Kwon I. Lee J. Chang S. H. Jung N. C. Lee B. J. Son G. H. Kim K. Lee K. H. (2006) BMAL1 shuttling controls transactivation and degradation of the CLOCK/BMAL1 heterodimer. Mol. Cell. Biol. 26, 7318–7330 16980631 10.1128/MCB.00337-06 PMC1592876

[28] Welsh D. K. Yoo S. H. Liu A. C. Takahashi J. S. Kay S. A. (2004) Bioluminescence imaging of individual fibroblasts reveals persistent, independently phased circadian rhythms of clock gene expression. Curr. Biol. 14, 2289–2295 15620658 10.1016/j.cub.2004.11.057 PMC3777438

[29] Cardone L. Hirayama J. Giordano F. Tamaru T. Palvimo J. J. Sassone-Corsi P. (2005) Circadian clock control by SUMOylation of BMAL1. Science 309, 1390–1394 16109848 10.1126/science.1110689

[30] Blanchard F. J. Collins B. Cyran S. A. Hancock D. H. Taylor M. V. Blau J. (2010) The transcription factor Mef2 is required for normal circadian behavior in *Drosophila*. J. Neurosci. 30, 5855–5865 20427646 10.1523/JNEUROSCI.2688-09.2010 PMC2876976

[31] Ha C. H. Kim J. Y. Zhao J. Wang W. Jhun B. S. Wong C. Jin Z. G. (2010) PKA phosphorylates histone deacetylase 5 and prevents its nuclear export, leading to the inhibition of gene transcription and cardiomyocyte hypertrophy. Proc. Natl. Acad. Sci. U.S.A. 107, 15467–15472 20716686 10.1073/pnas.1000462107 PMC2932618

[32] O'Neill J. S. Maywood E. S. Chesham J. E. Takahashi J. S. Hastings M. H. (2008) cAMP-dependent signaling as a core component of the mammalian circadian pacemaker. Science 320, 949–953 18487196 10.1126/science.1152506 PMC2735813

